# Subdiffractional focusing and guiding of polaritonic rays in a natural hyperbolic material

**DOI:** 10.1038/ncomms7963

**Published:** 2015-04-22

**Authors:** S. Dai, Q. Ma, T. Andersen, A. S. Mcleod, Z. Fei, M. K. Liu, M. Wagner, K. Watanabe, T. Taniguchi, M. Thiemens, F. Keilmann, P. Jarillo-Herrero, M. M. Fogler, D. N. Basov

**Affiliations:** 1 Department of Physics, University of California, San Diego, La Jolla, California 92093, USA; 2 Department of Physics, Massachusetts Institute of Technology, Cambridge, Massachusetts 02215, USA; 3 Department of Physics, Stony Brook University, Stony Brook, New York 11794, USA; 4 National Institute for Materials Science, Namiki 1-1, Tsukuba, Ibaraki 305-0044, Japan; 5 Department of Chemistry and Biochemistry, University of California, San Diego, La Jolla, California 92093, USA; 6 Ludwig-Maximilians-Universität and Center for Nanoscience, 80539 München, Germany

## Abstract

Uniaxial materials whose axial and tangential permittivities have opposite signs are referred to as indefinite or hyperbolic media. In such materials, light propagation is unusual leading to novel and often non-intuitive optical phenomena. Here we report infrared nano-imaging experiments demonstrating that crystals of hexagonal boron nitride, a natural mid-infrared hyperbolic material, can act as a ‘hyper-focusing lens' and as a multi-mode waveguide. The lensing is manifested by subdiffractional focusing of phonon–polaritons launched by metallic disks underneath the hexagonal boron nitride crystal. The waveguiding is revealed through the modal analysis of the periodic patterns observed around such launchers and near the sample edges. Our work opens new opportunities for anisotropic layered insulators in infrared nanophotonics complementing and potentially surpassing concurrent artificial hyperbolic materials with lower losses and higher optical localization.

## Abstract (web summary)

Hexagonal boron nitride has many interesting properties, including a natural hyperbolic dispersion, making it attractive for nanophotonic applications. Here, Dai *et al*. show that metallic disks under the material launch phonon–polaritons, turning it into a hyper-focusing lens.

## Graphical abstract

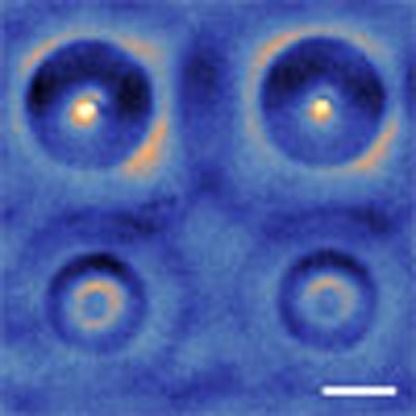

One of the primary goals of nanophotonics is concentration of light on scales shorter than the free-space wavelength *λ*. According to the general principles of Fourier optics, this is only possible provided electromagnetic modes of large tangential momenta *k*_t_>*ω*/(2*π*), normally evanescent, are nonetheless able to reach the focal plane (the *x*–*y* plane). Here *ω=λ*^−1^ is the measure of frequency common in spectroscopy and 
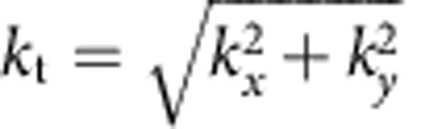
. In devices known as superlenses^1^,^2^,^3^,^4^,^5^,^6^, this requirement is realized via resonant tunneling between the opposite sides of the structure. However, the tunneling is very sensitive to damping, for example, the magnitude of the imaginary part of the permittivity *ɛ* of the superlens material^7^. The largest characteristic momentum that can pass through a superlens of thickness *d* can be found from the relation 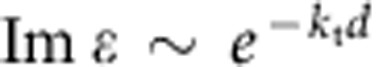. In this regard, hyperbolic media (HM)^8^,^9^ promise a significant advantage as they support large-*k* hyperbolic polaritons that remain propagating rather than evanescent, so that the condition on damping is much softer (see below). The unusual properties of hyperbolic polaritons in HM^8^,^9^,^10^,^11^,^12^,^13^,^14^,^15^,^16^,^17^,^18^,^19^,^20^ stem from the dispersion of these modes that is described by the equation of a hyperboloid:


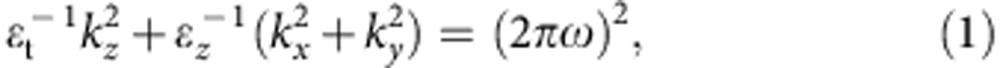


where *ɛ*_*z*_ and *ɛ*_t_≡*ɛ*_*x*_=*ɛ*_*y*_ are the axial and tangential permittivities, respectively. The hyperboloid is single-sheeted if *ɛ*_*z*_>0, *ɛ*_t_<0 (type II) and two-sheeted if *ɛ*_*z*_<0, *ɛ*_t_>0 (type I), see Fig 1a,b, respectively. In both cases, the slope of the propagation (group velocity) direction, which is orthogonal to the dispersion surface, asymptotically approaches


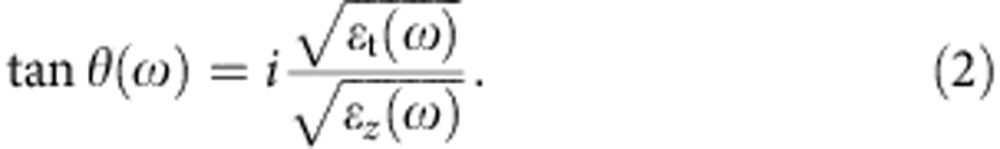


The condition for achieving super-resolution is Im *k*_*z*_*d*=(*k*_t_*d*)Im tan *θ*∼1. Hence, admissible Im *ɛ*_*z*_, Im *ɛ*_t_ scale algebraically rather than exponentially with the resolution 
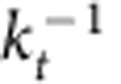
.

Directional propagation of hyperbolic polaritons along ‘resonance cones' of apex angle *θ* has been observed in a magnetized plasma^21^,^22^, which behaves as a natural HM in the microwave domain. A major resurgence of interest to HM was prompted by their discussion in the context of artificial materials (metamaterials)^23^,^24^. Examples of such hyperbolic metamaterials include microstrip arrays, where directional propagation and focusing of hyperbolic polaritons have been experimentally observed^25^,^26^. Directional optical beams have been studied in planar^25^,^26^,^27^,^28^ and curved^12^,^29^ metamaterials made of alternating layers of metals and semiconductors. The work on non-planar structures^12^,^29^ was motivated by theoretical proposals of a hyperlens^30^,^31^,^32^, a device in which directional beams outgoing from a subdiffractional source enable optical magnification. However, improvement over the diffraction limit has so far been severely impeded by losses in constituent metals and imperfections of nanofabrication.

Recent work^33^,^34^ identified hexagonal boron nitride (hBN) as a low-loss natural HM in the mid-infrared domain. This layered insulator has emerged as a premier substrate or a spacer for van der Waals heterostructures^35^,^36^. Light atomic masses, strong anisotropy and the polar band between B and N yield prominent optical phonon modes that create two widely separated stop-bands—spectral intervals where one of the principal values of the dielectric tensor is negative^33^,^34^,^37^. The upper band comprises *ω*=1,370–1,610 cm^−1^ where the real part of *ɛ*_t_ (the in-plane permittivity) is negative while that of *ɛ*_*z*_ is positive. In the lower band spanning *ω*=746–819 cm^−1^, the signs of the permittivity components are reversed. Thus, the out-of-plane crystal vibrations enable the type I hyperbolic response, whereas the in-plane ones accounts for the type II behaviour. The momentum-frequency dispersion surface for the hyperbolic polaritons of the upper band resembles a ‘butterfly' (Fig. 1c) composed of individual hyperbolas sketched in Fig. 1a. It can be contrasted with the flat dispersion surfaces of longitudinal phonons typical for isotropic materials. Effectively, in hBN the longitudinal phonons are hybridized with the transverse ones by quasi-static Coulomb interaction mediated by large-*k* photons^38^. Because the hyperbolic response in hBN originates from the anisotropic phonons, in the following, the large-*k* hyperbolic polaritons are referred to as hyperbolic phonon polaritons (HP^2^).

## Results

### Subdiffractional focusing and imaging through hBN

In our experiments, efficient excitation and detection of HP^2^ in hBN are accomplished with the help of optical antenna structures^39^,^40^. The antennas concentrate electric field and bridge the large momentum mismatch between the free-space photons and the HP^2^. In our previous work^33^, we used for this purpose a sharp tip of an atomic force microscope (AFM) incorporated in our scattering-type scanning near-field optical microscopy (s-SNOM) apparatus (Methods). Here, we additionally demonstrate the antenna and polariton-launching capabilities of Au disks patterned on a SiO_2_ substrate. The AFM topography image in Fig. 2a depicts Au disks of diameters (top to bottom) 1,000, 500 and 200 nm and thickness of about 50 nm. After the subsequent deposition of hBN crystals of thickness *d*=100–1,060 nm and lateral sizes up to 10 μm, these Au disks become encapsulated between hBN and SiO_2_. The hBN crystal remains essentially flat, as verified by AFM. Below we present experimental results demonstrating that interaction of these disks with an incident infrared beam excites polaritons that travel across hBN and produce specific contrast patterns at the other surface. We show that the observed dependence of the near-field images on the frequency and hBN thickness is the result of directional propagation of the polaritons along conical surfaces with frequency-tunable apex angle given by Equation (2). Thus, hBN may emerge as a new standard bearer for mid-infrared nanophotonics by enabling devices for deeply subdiffractional propagation, focusing and imaging with tunable characteristics.

Representative s-SNOM imaging data are shown in Fig. 2. Figure 2b depicts an s-SNOM scan taken at the top surface of hBN of thickness *d*=395 nm at frequency *ω*=1,515 cm^−1^ (*λ*=6.6 μm). Here we plot the third harmonics of the scattering amplitude *s*(*ω*) (Methods). In this image, each Au disk is surrounded by a series of concentric ‘hot rings' of strongly enhanced nano-infrared contrast. The diameters of all the disks are much smaller than *λ* (see also Fig. 2a), the smallest one being 200 nm=*λ*/33. The diameters of the hot rings can be larger, smaller or equal to those of the disks. The spacing between adjacent hot rings in the same sample increases with the infrared frequency but decreases with the sample thickness. We stress that images displayed in Fig. 2b could only be detected if the infrared wavelength falls inside the hyperbolic spectral regions. Outside of the hBN stop bands, no hot rings can be identified by the s-SNOM. In fact, the entire image is homogeneous, comprised of nothing but random noise, as illustrated by Fig. 2d for *ω*=1,740 cm^−1^ (*λ*=5.7 μm).

We now elaborate on the formation of images in Fig. 2 recorded with our s-SNOM apparatus with the help of a model of HP^2^ propagation through a slab of hBN (Fig. 3a,c,d). Consider a perfectly thin metallic disk sandwiched between a slab of a HM of thickness *d* and an isotropic dielectric substrate. The system is subject to a uniform electric field of frequency *ω* and amplitude *E*_0_ in the *x* direction. An approximate solution for the total field in this system can be found analytically (Supplementary Note 1). The corresponding distributions of the *z*-component of the field *E*_*z*_(*x*, *y*, *z*) in the two cross-sections, *y*=0 (the vertical symmetry plane) and *z*=*d*−0 (just below the top surface of the hBN slab), are illustrated in Fig. 3c. These plots are computed for three representative radii of the disk using permittivity values at *ω*=1,515 cm^−1^. The plots demonstrate a series of concentric high-intensity rings on the top surface, very similar to the data in Fig. 2b. The interpretation (Fig. 3a,c) is straightforward: the external field polarizes the disk, which perturbs the adjacent HM (hBN in our case) and launches polaritons. The HP^2^ emission occurs predominantly at disk edges due to the high concentration of electric field therein. Polaritonic rays propagate across the slab, maintaining a fixed angle *θ* with respect to the *z* axis: the ‘resonance cone' direction^18^,^21^,^22^,^25^,^26^,^27^,^28^. Upon reaching the other slab surface, they undergo a total internal reflection with the reflected cone extending toward the bottom surface. The process repeats until eventually the field vanishes because of radial spreading and/or damping. The role of the s-SNOM tip in imaging experiments in Fig. 2 is to out-couple HP^2^ fields at the top surface (Fig. 3a). The observed s-SNOM signal is roughly proportional to the amplitude of the electric field immediately above the slab *E*_*z*_(*z*=*d*=0). (Note that it is related to the field just inside the slab by a constant factor, *E*_*z*_(*z*=*d*+0)=*ɛ*_*z*_(*ω*)*E*_*z*_(*z*=*d*−0).)

The above model of image formations via HP^2^ yields a number of quantitative predictions that are in accord with our observations. The scenario of oblique propagation implies that upon each roundtrip across the slab, the excitation front returns to the same surface displaced radially by the distance


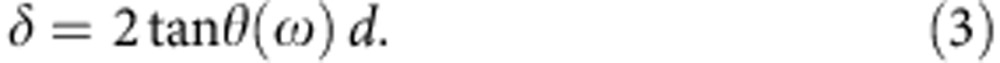


Accordingly, the radii of the ‘hot rings' at the top surface of the slab are given by


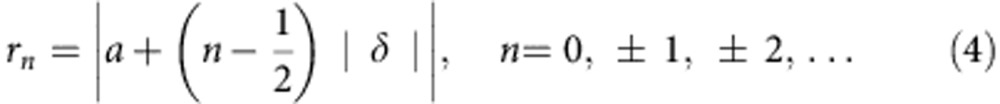


where *a* is the disk radius. The intensity of the rings is expected to decrease with |*n*|. Consistent with this formula, the smallest rings in Fig. 3c have the radius *r*_0_=|*a*−|δ|/2|. Particularly interesting is the case where the innermost ring shrinks to a single bright spot, *r*_0_=0. Experimentally, we observed spots of diameter 200 nm (the full width at half maximum, see Supplementary Note 1), which corresponds to *λ*/33 for Fig. 2b (top). Focal spots of similar size 185–210 nm were observed in all other hBN crystals, with the thickness up to 1,050 nm (Supplementary Fig. 5).

A proposal for focusing of electromagnetic radiation via resonance-cone propagation in hyperbolic media was theoretically discussed in the context of magneto-plasmas^21^. Experimental confirmation of this idea in an artificial hyperbolic multi-layer was reported where *λ*/6 focusing was deduced from examining the pattern of a polymerized photoresist behind a two-slit polaritonic launcher^26^. Here, using a natural hyperbolic slab (hBN crystal), we demonstrated the *λ*/33 focusing in both spatial directions via out-coupling of polaritons with the infrared nano-probe. We stress that a distinction should be made between ‘focusing' and ‘imaging.' Focusing devices can be of both imaging and non-imaging type^41^ and both are important in applications. Our hBN device (Fig. 3a) is an example of the latter.

Continuing with the verifiable predictions of our model, we note that Equations (3) and (4) indicate that the slope tan *θ* of the resonance cone is uniquely related to the radii of the hot rings (Fig. 3a). To test this prediction, we analysed the images collected from samples of different hBN thicknesses and different Au disk diameters. For each of these, we determined the radius *r*_1_ of the first-order ring and computed |tan *θ*|=(*r*_1_−*a*)/*d* as a function of the infrared frequency (Fig. 3a). As shown in Fig. 3b, all the data collapse toward a single smooth curve computed from Equation (2) using optical constants of hBN from ref. 33. Yet another prediction of the model: the polaritonic rays travel along the *z* axis provided that *ɛ*_t_(*ω*) and therefore *θ*(*ω*) are vanishingly small. This condition is satisfied at *ω*=1,610 cm^−1^ (Fig. 2c) where we observe almost 1:1 images of Au disks. Similar behaviour was observed when instead of the disks more complicated metallic shapes were imaged (Supplementary Fig. 5). Thus, the totality of our data establishes the notion of directional propagation of HP^2^ in hBN over macroscopic distances with a frequency-tunable slope (Fig. 3b).

### Real-space imaging of multiple guided polaritons in hBN

The outlined real-space picture has a counterpart in its conjugate momentum space. Mathematically, the resonance cones in the real space are coherent superpositions of an infinite number of polariton modes of a slab. Such modes are characterized by quantized momenta, *k*_*z*,*l*_=(*π*/*d*)(*l*+*α*), labelled by integer index *l*^33^. Here *α*∼1 (in general, *ω*-dependent) quantifies the phase shift acquired at the total internal reflection from the slab surfaces. Per Equation (1), the tangential momenta of these modes are also quantized,


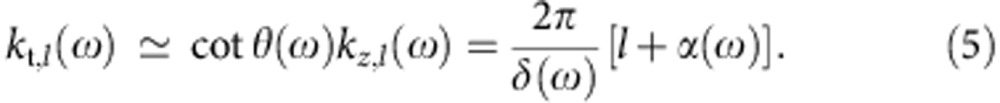


**Figure 1:**
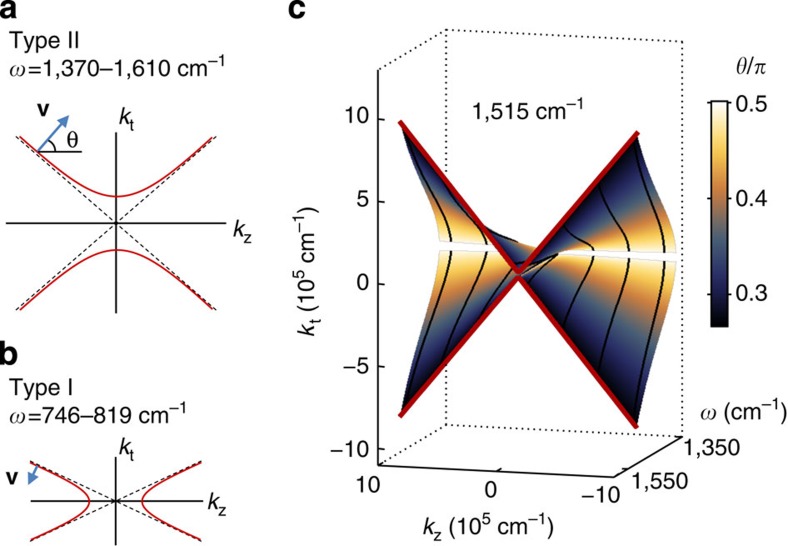
Hyperbolic dispersion of hBN. (**a**) A sketch of the isofrequency curves for a type II HM, which is realized in the upper stop-band of hBN. The arrow indicates the polariton group velocity. (**b**) A similar sketch for the type I case, which is realized in the hBN lower stop-band. (**c**) The calculated dispersion surface of hBN polaritons. The axes are the tangential momentum (*k*_t_), the axial momentum (*k*_*z*_) and the frequency (*ω*, ranging from 1,370 to 1,515 cm^−1^). The colour represents the propagation angle *θ*. The constant-frequency cut *ω*=1,515 cm^−1^ is shown by the red line, to emphasize similarity with **a**. The dispersion of polaritons in a finite-thickness crystal (*d*=105 nm) is shown by the black lines to clarify their relation to Fig. 4a.

**Figure 2:**
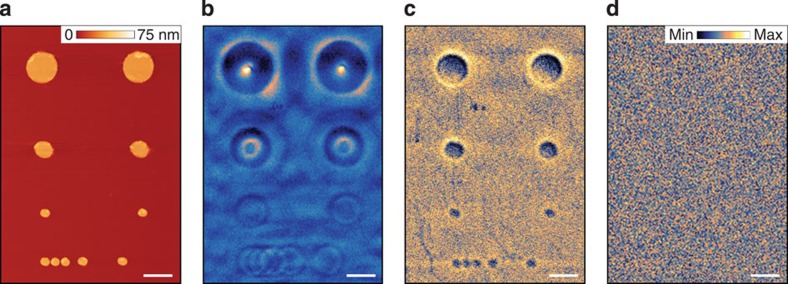
Sub-diffractional focusing and imaging through an hBN crystal. (**a**) An AFM image of Au disks defined lithographically on SiO_2_/Si substrate before hBN transfer. (**b**) Near-field amplitude image of the top surface of a 395-nm-thick hBN at infrared laser frequency *ω*=1,515 cm^−1^ (*λ*=6.6 μm). The observed ‘hot rings' are concentric with the Au discs. (**c**) Near-field image of the same sample as in **b** at *ω*=1,610 cm^−1^ (*λ*=6.2 μm) where polaritons propagate almost vertically. (**d**) Near-field image of the same sample at *ω*=1,740 cm^−1^ (*λ*=5.7 μm) showing complete homogeneity and lack of any distinct features. The colour scales for **b**–**d** are indicated in **d**. The scale bars in all panels are 1 μm long.

In the last step, we have applied Equation (3). For illustration, the dispersion curves of such guided modes in the upper stop-band of hBN of thickness 105 nm are shown in Fig. 1c, where they are overlaid on the dispersion surface of bulk hBN. The same curves are replotted as *ω* vs *k*_t_ in Fig. 4a. In Fig. 4b the dispersion curves of the guided modes of lower stop-band are shown. An intriguing aspect of these curves is that their slope ∂*ω*/∂*k*_t_ is positive (negative) in the upper (lower) band. This sign difference is a consequence of the opposite direction of the group velocity vector for the type I and type II cases, cf. Fig. 1a,b. Central to the connection between the resonance cones in the real space and the quantized momenta in the *k*-space is that these momenta form an equidistant sequence of period Δ*k*_t_=*k*_t,*l*+1_−*k*_t,*l*_=2*π*/*δ*. Therefore, if several guided modes are excited simultaneously by a source, their superposition would produce beats with period 2*π*/Δ*k*_t_ in real space. This is precisely the spacing *δ* between periodic revivals of the ‘hot rings' (Equation (3) and Fig. 2). Thus, the multi-ring images and the existence of higher-order guided modes are complementary manifestations of the same fundamental physics. In our previous work^33^, we reported nano-imaging and nano-spectroscopic study of the lowest-momentum guided mode *l*=0 in hBN crystals. Below we present new results documenting the first observation of the higher-order (up to three) guided modes in such materials by direct nano-infrared imaging.

To map the dispersion of HP^2^, we utilized hBN crystals on SiO_2_ substrate without any intervening metallic disks (Methods). Here the sharp tip of the s-SNOM serves as both the emitter and the detector of the polariton waves on the open surface of the hBN. As the tip is scanned toward the sample edge, distinct variations in the detected scattering amplitude *s*(*ω*) are observed. Such variations are caused by passing over minima and maxima of the standing waves created by interference of the polaritons launched by the tip and their reflections off the sample edges (Fig. 5a). Representative data for the upper stop-band (the type II hyperbolic region) are shown in Fig. 5b–f, where we plot *s*(*ω*) at various infrared frequencies. Specifically, the image presented in Fig. 5b exhibits oscillations with the period ∼1 μ m extending parallel to the edge of a 31-nm-thick hBN crystal. While these oscillations are similar to those reported previously^33^, a high-resolution scan performed very close to the edge (the olive square) reveals additional oscillations occurring on a considerably shorter scale: down to hundreds of nanometres (Fig. 5c–e). Similar results have been obtained using many other samples. For example, Fig. 5f also shows short-scale oscillations near the edges co-existing with longer-range oscillations further away from the edge in the data collected for a thicker hBN crystal (*d*=105 nm).

To analyse the harmonic content of the measured *s*(*ω*) quantitatively, we used the spatial Fourier transform (FT). An example shown in Fig. 5h is the FT of the line trace α from Fig. 5g. The three dominant peaks in the FT are marked with β' (blue), γ' (magenta) and ζ' (olive). These peaks have been deemed statistically significant and their positions *k*_β_, *k*_γ_ and *k*_ζ_ have been recorded for each of the traces studied. We reasoned that including additional weaker peaks into consideration may be unwarranted at this stage. Indeed, the gross features in the real-space trace α exceeding the noise level of ∼1 a.u. are accounted for by oscillations in the three partial traces β, γ and ζ, which are obtained by the inverse FT of the shaded regions in Fig. 5h.

The remaining step in the analysis is to establish the connection of thus determined momenta *k*_β_, *k*_γ_ and *k*_ζ_ and the momenta *k*_t*,l*_ of the guided modes, Equation (5). This requires more care than in prior studies of single-mode waves in two-dimensional materials^33^,^42^,^43^,^44^. The interference patterns near the edge can be created by various combinations of the tip-launched waves (labeled by *l*) and edge-reflected waves (labeled by *r*). The total momentum of a particular combination is *k*_t,*l*_+*k*_t,*r*_. If the mode index is conserved, *l*=*r*, the set of possible periods narrows down to 2*k*_t,*l*_. This is consistent with our data obtained for several infrared frequencies (Fig. 4a), where the symbols indicate *k*_β_, *k*_γ_ and *k*_ζ_. These data are in a quantitative agreement with the calculated dispersion curves for the *l*=0, 1 and 2 polariton-guided waves in the upper stop-band. The analysis of polariton propagation length^33^ shows that the loss factor is as low as *γ*∼0.03 (Supplementary Fig. 4). Dispersion mapping in the lower band (746–819 cm^−1^) where no monochromatic lasers are available is discussed in Supplementary Fig. 3. Broad-band lasers used in an independent study by Li *et al*. have allowed to demonstrate focusing behaviour of hBN in this challenging frequency region^45^.

## Discussion

**Figure 3:**
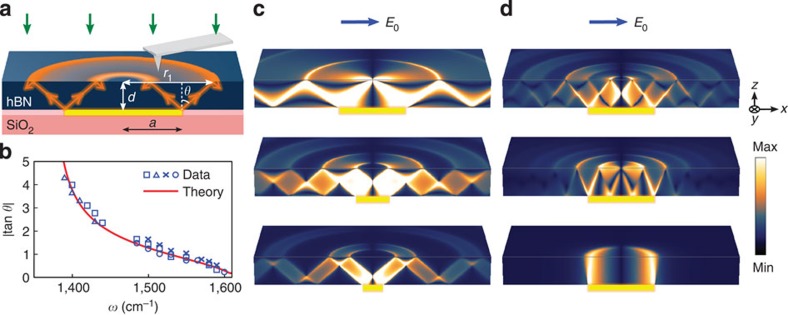
Image formation. (**a**) Imaging schematics. Under infrared illumination (green arrow), the polaritons were launched by the Au disk edges and propagate towards the hBN top surface where the near-field images were recorded via the back-scattered infrared beam (green arrow). The propagation angle *θ* can be inferred from the hot ring radius *r*_1_, hBN thickness *d* and disk radius *a*. (**b**) The tangent of the propagation angle *θ* derived from imaging data for different hBN samples (symbols) and from Equation (2) (solid line). Squares, triangles, crosses and dots indicate data from hBN samples with thickness *d*=395, 984, 270 and 1,060 nm, respectively. (**c**) The distribution of the *z*-component of the electric field in the analytical model (see text). The hot rings on the surfaces appear as a result of multiple reflections of polaritons launched at the disk edges. The ratio *a*/|δ|=0.5, 0.25, 0.15 decreases from top to bottom. In the top picture, the smallest ring shrinks to a focal point. The blue arrow indicates the direction of electric field *E*_0_ in simulation. (**d**) Similar to **b** for *a*/*d*=1.12 and (top to bottom) |tan *θ*|=0.75, 0.375 and 0.01.

**Figure 4:**
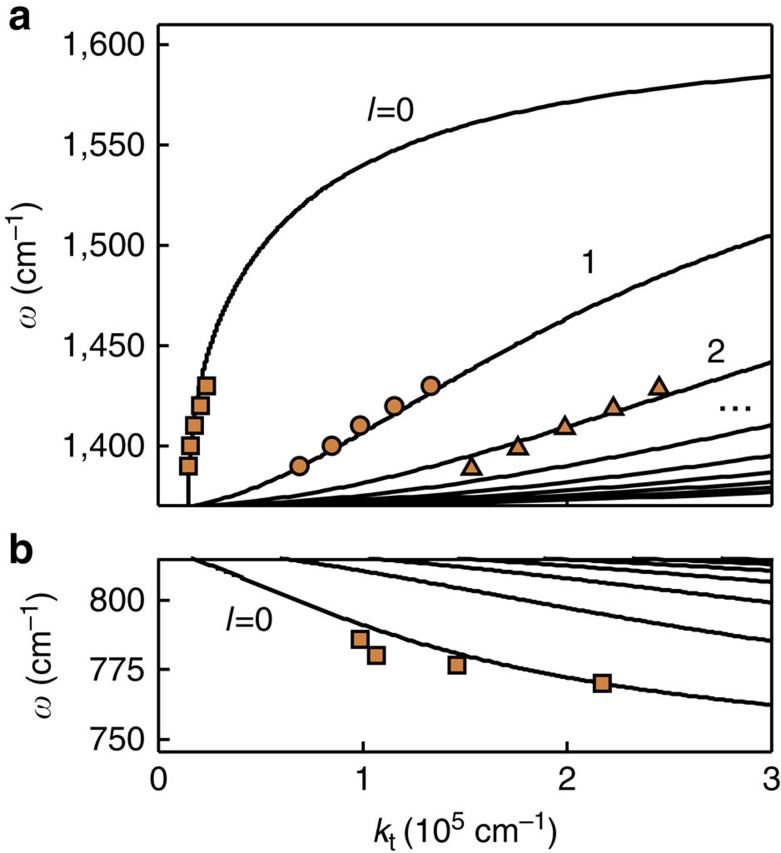
Polariton frequency (*ω*) – in-plane momentum (*k*_t_) dispersion relation for hBN. (**a**) The dispersion curves from Fig. 1c replotted as frequency (*ω*) versus in-plane momenta (*k*_t_). The experimental data (squares) are obtained from the polariton reflection images near the sample edges (Fig. 5). (**b**) Same as **a** for the lower hBN stop-band (Supplementary Fig. 3). Thickness of hBN: 105 nm.

**Figure 5:**
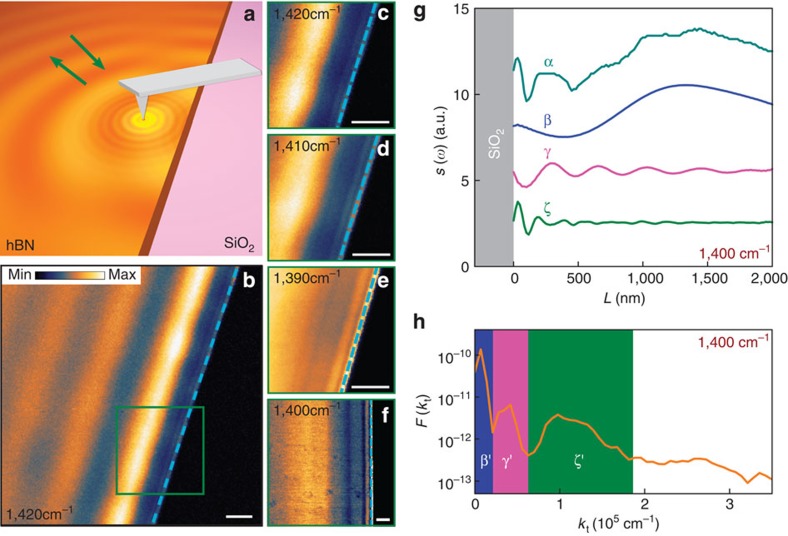
Imaging of polariton waveguide modes near the hBN edges. (**a**) Experimental schematic is similar to Fig. 3a except that imaging here is performed near the edge of an unpatterned sample. (**b**) Near-field amplitude image measured at 1,420 cm^−1^. The olive square indicates the area whose expanded view is shown in **c**–**e**). (**c**–**e**) Near-field image of the area marked in **b** at several frequencies. hBN thickness in **b**−**e**: 31 nm. (**f**) Near-field image of 105-nm-thick hBN at 1,400 cm^−1^. The cyan dashed lines in **b**−**f** indicate the hBN edges. Scale bar in **b**−**f**, 300 nm. (**g**) Line traces perpendicular to the hBN edge. Trace α was extracted from the image in **f**. Traces β, γ and ζ were obtained from the Fourier analysis of the trace α as described in the text. (**h**) The Fourier transform of trace α in **g**.

Data presented in Figs 2, 3, 4, 5 demonstrate launching, long-distance waveguiding transport and focusing of electromagnetic energy in thin crystals of hBN. These phenomena are enabled by directional propagation of large-momentum polariton beams in this natural hyperbolic material. The sharpness of the attained focusing, *λ*/33 at distances up to *λ*/6 (Supplementary Fig. 5), in units of the free-space wavelength, surpasses all prior realizations of superlenses and hyperlenses. Remarkably, a simple addition of a circular metallic launcher transforms an hBN crystal into a powerful focusing^19^ device! The analysis presented in Supplementary Note 1 (Supplementary Equation 10) indicates that the size of the focal spot in our system is limited by the finite thickness ∼50 nm of Au disks. By using thinner disks, say 20 nm thick, one should be able to achieve focal spots as small as ∼*λ*/10^2^, comparable to the spatial resolution of our nano-infrared apparatus. A fundamental advantage of using natural rather than artificial hyperbolic materials is the magnitude of the upper momentum cutoff. In a natural material such as hBN, this cutoff is ultimately set by interatomic spacing thus immensely enhancing the spatial resolution. In addition, we have shown that hBN can serve as a multi-mode waveguide for polaritons with excellent figure of merit: loss factor as small as *γ*∼0.03. These characteristics exceed the benchmarks^46^,^47^,^48^ of current metal-based plasmonics and metamaterials. The physics behind this fundamental advantage of phonon polaritons over plasmons in conducting media is in the absence of electronic losses in insulators. Applications of hBN for non-imaging focusing devices^41^, subdiffractional waveguides and nanoresonators^34^ readily suggest themselves^45^. Combining such elements together may lead to development of sophisticated nanopolaritonic circuits.

## Methods

### Experimental setup

The nano-imaging and nano-FTIR experiments described in the main text were performed at UCSD using a commercial s-SNOM (www.neaspec.com). The s-SNOM is based on a tapping-mode AFM illuminated by monochromatic quantum cascade lasers (QCLs) (www.daylightsolutions.com) and a broad-band laser source utilizing the difference frequency generation (www.lasnix.com)^49^. Together, these lasers cover a frequency range of 700–2,300 cm^−1^ in the mid-infrared. The nanoscale near-field images were registered by pseudo-heterodyne interferometric detection module with AFM tapping frequency and amplitude around 250 kHz and 60 nm, respectively. To obtain the background-free images, the s-SNOM output signal used in this work is the scattering amplitude *s*(*ω*) demodulated at the *n*th harmonics of the tapping frequency. We chose *n*=3 in this work.

### Sample fabrication

Silicon wafers with 300-nm-thick SiO_2_ top layer were used as substrates for all the samples. The Au patterns of various lateral shapes and 50-nm thickness were fabricated on these wafers by electron beam lithography. The hBN microcrystals of various thicknesses were exfoliated from bulk samples synthesized under high pressure^50^. Such microcrystals were subsequently mechanically transferred onto either patterned or unpatterned parts of the substrates.

## Author contributions

All the authors were involved in designing the research, performing the research and writing the paper.

## Additional information

**How to cite this article**: Dai, S. *et al*. Subdiffractional focusing and guiding of polaritonic rays in a natural hyperbolic material. *Nat. Commun*. 6:6963 doi: 10.1038/ncomms7963 (2015).

## Supplementary Material

Supplementary Information Supplementary Figures 1-5, Supplementary Notes 1-2 and Supplementary References.

## References

[b1] Pendry J. B. Negative refraction makes a perfect lens. Phys. Rev. Lett. 85, 3966–3969 (2000). 11041972 10.1103/PhysRevLett.85.3966

[b2] Fang N., Lee H., Sun C. & Zhang X. Sub-diffraction-limited optical imaging with a silver superlens. Science 308, 534–537 (2005). 15845849 10.1126/science.1108759

[b3] Taubner T., Korobkin D., Urzhumov Y., Shvets G. & Hillenbrand R. Near-field microscopy through a SiC superlens. Science 313, 1595 (2006). 16973871 10.1126/science.1131025

[b4] Zhang X. & Liu Z. Superlenses to overcome the diffraction limit. Nat. Mater. 7, 435–441 (2008). 18497850 10.1038/nmat2141

[b5] Smolyaninov I. I., Hung Y. J. & Davis C. C. Magnifying superlens in the visible frequency range. Science 315, 1699–1701 (2007). 17379804 10.1126/science.1138746

[b6] Kehr S. C. . Near-field examination of perovskite-based superlenses and superlens-enhanced probe-object coupling. Nat. Commun. 2, 249 (2011). 21427720 10.1038/ncomms1249 PMC3072079

[b7] Smith D. R. . Limitations on subdiffraction imaging with a negative refractive index slab. Appl. Phys. Lett. 82, 1506–1508 (2003).

[b8] Poddubny A., Iorsh I., Belov P. & Kivshar Y. Hyperbolic metamaterials. Nat. Photonics 7, 948–957 (2013).

[b9] Guo Y., Newman W., Cortes C. L. & Jacob Z. Applications of hyperbolic metamaterial substrates. Adv. Optoelectron. 2012, 452502 (2012).

[b10] Hoffman A. J. . Negative refraction in semiconductor metamaterials. Nat. Mater. 6, 946–950 (2007). 17934463 10.1038/nmat2033

[b11] Yao J. . Optical negative refraction in bulk metamaterials of nanowires. Science 321, 930 (2008). 18703734 10.1126/science.1157566

[b12] Liu Z., Lee H., Xiong Y., Sun C. & Zhang X. Far-field optical hyperlens magnifying sub-diffraction-limited objects. Science 315, 1686 (2007). 17379801 10.1126/science.1137368

[b13] Yang X., Yao J., Rho J., Yin X. & Zhang X. Experimental realization of three-dimensional indefinite cavities at the nanoscale with anomalous scaling laws. Nat. Photonics 6, 450–454 (2012).

[b14] Argyropoulos C., Estakhri N. M., Monticone F. & Alu A. Negative refraction, gain and nonlinear effects in hyperbolic metamaterials. Opt. Express 21, 15037–15047 (2013). 23787691 10.1364/OE.21.015037

[b15] Biehs S. A., Tschikin M. & Ben-Abdallah P. Hyperbolic metamaterials as an analog of a blackbody in the near field. Phys. Rev. Lett. 109, 104301 (2012). 23005292 10.1103/PhysRevLett.109.104301

[b16] Noginov M. A. . Controlling spontaneous emission with metamaterials. Opt. Lett. 35, 1863–1865 (2010). 20517443 10.1364/OL.35.001863

[b17] Guo Y., Cortes C. L., Molesky S. & Jacob Z. Broadband super-Planckian thermal emission from hyperbolic metamaterials. Appl. Phys. Lett. 101, 131106 (2012).

[b18] Jacob Z., Smolyaninov I. I. & Narimanov E. E. Broadband Purcell effect: radiative decay engineering with metamaterials. Appl. Phys. Lett. 100, 181105 (2012).

[b19] Smith D. R., Schurig D., Mock J. J., Kolinko P. & Rye P. Partial focusing of radiation by a slab of indefinite media. Appl. Phys. Lett. 84, 2244–2246 (2004).

[b20] Vinogradov A. P., Dorofeenko A. V. & Nechepurenko I. A. Analysis of plasmonic Bloch waves and band structures of 1D plasmonic photonic crystals. Metamaterials 4, 181–200 (2010).

[b21] Fisher R. K. & Gould R. W. Resonance cones in the field pattern of a short antenna in anisotropic plasma. Phys. Rev. Lett. 22, 1093–1095 (1969).

[b22] Levine B., Greene G. J. & Gould R. W. Focusing resonance cones. Phys. Fluids 21, 1116–1119 (1978).

[b23] Lindell I. V., Tretyakov S. A., Nikoskinen K. I. & Ilvonen S. BW media-media with negative parameters, capable of supporting backward waves. Microw. Opt. Technol. Lett. 31, 129–133 (2001).

[b24] Smith D. R. & Schurig D. Electromagnetic wave propagation in media with indefinite permittivity and permeability tensors. Phys. Rev. Lett. 90, 077405 (2003). 12633273 10.1103/PhysRevLett.90.077405

[b25] Siddiqui O. & Eleftheriades G. V. Resonance-cone focusing in a compensating bilayer of continuous hyperbolic microstrip grids. Appl. Phys. Lett. 85, 1292 (2004).

[b26] Ishii S., Kildishev A. V., Narimanov E., Shalaev V. M. & Drachev V. P. Sub-wavelength interference pattern from volume plasmon polaritons in a hyperbolic medium. Laser Photonics Rev. 7, 265–271 (2013).

[b27] Thongrattanasiri S. & Podolskiy V. A. Hypergratings: nanophotonics in planar anisotropic metamaterials. Opt. Lett. 34, 890–892 (2009). 19340161 10.1364/ol.34.000890

[b28] Ishii S., Drachev V. P. & Kildishev A. V. Diffractive nanoslit lenses for subwavelength focusing. Opt. Commun. 285, 3368–3372 (2012).

[b29] Rho J. . Spherical hyperlens for two-dimensional sub-diffractional imaging at visible frequencies. Nat. Commun. 1, 143 (2010). 21266993 10.1038/ncomms1148

[b30] Jacob Z., Alekseyev L. V. & Narimanov E. Optical hyperlens: far-field imaging beyond the diffraction limit. Opt. Express 14, 8247–8256 (2006). 19529199 10.1364/oe.14.008247

[b31] Salandrino A. & Engheta N. Far-field subdiffraction optical microscopy using metamaterial crystals: theory and simulations. Phys. Rev. B 74, 075103 (2006).

[b32] Lu D. & Liu Z. Hyperlenses and metalenses for far-field super-resolution imaging. Nat. Commun. 3, 1205 (2012). 23149749 10.1038/ncomms2176

[b33] Dai S. . Tunable phonon polaritons in atomically thin van der waals crystal of boron nitride. Science 343, 1125–1129 (2014). 24604197 10.1126/science.1246833

[b34] Caldwell J. D. . Sub-diffraction, volume-confined polaritons in the natural hyperbolic material, hexagonal boron nitride. Nat. Commun. 5, 5221 (2014). 25323633 10.1038/ncomms6221

[b35] Geim A. K. & Grigorieva I. V. Van der Waals heterostructures. Nature 499, 419–425 (2013). 23887427 10.1038/nature12385

[b36] Fogler M. M., Butov L. V. & Novoselov K. S. High-temperature super-fluidity with indirect excitons in van der Waals heterostructures. Nat. Commun. 5, 4555 (2014). 25065343 10.1038/ncomms5555

[b37] Xu X. G. . One-dimensional surface phonon polaritons in boron nitride nanotubes. Nat. Commun. 5, 4782 (2014). 25154586 10.1038/ncomms5782

[b38] Tarkhanyan R. & Uzunoglu N. Radiowaves and polaritons in anisotropic media: uniaxial semiconductors Wiley: Hoboken (2006).

[b39] Novotny L. & Hecht B. Principles Of Nano-Optics Cambridge Univ. Press (2006).

[b40] Atkin J. M., Berweger S., Jones A. C. & Raschke M. B. Nano-optical imaging and spectroscopy of order, phases, and domains in complex solids. Adv. Phys. 61, 745–842 (2012).

[b41] Chaves J. Introduction to Nonimaging Optics CRC Press (2008).

[b42] Chen J. . Optical nano-imaging of gate-tunable graphene plasmons. Nature 487, 77–81 (2012). 22722861 10.1038/nature11254

[b43] Fei Z. . Gate-tuning of graphene plasmons revealed by infrared nano-imaging. Nature 487, 82–85 (2012). 22722866 10.1038/nature11253

[b44] Woessner A. . Highly confined low-loss plasmons in graphene-boron nitride heterostructures. Nat. Mater. 487, 82–85 (2015). 10.1038/nmat4169 25532073

[b45] Li P. . Hyperbolic Phonon-polaritons in Boron Nitride for near-field optical imaging. (2015) http://arxiv.org/abs/1502.04093. 10.1038/ncomms8507 PMC4491815 26112474

[b46] Boltasseva A. & Atwater H. Low-loss plasmonic metamaterials. Science 331, 290–291 (2011). 21252335 10.1126/science.1198258

[b47] Tassin P., Koschny T., Kafesaki M. & Soukoulis C. A comparison of graphene, superconductors and metals as conductors for metamaterials and plasmonics. Nat. Photonics 6, 259–264 (2012).

[b48] Khurgin J. B. & Boltasseva A. Reflecting upon the losses in plasmonics and metamaterials. MRS Bull. 37, 768–779 (2012).

[b49] Keilmann F. & Amarie S. Mid-infrared frequency comb spanning an octave based on an Er fiber laser and difference-frequency generation. J. Infrared Millimeter Terahertz Waves 33, 479–484 (2012).

[b50] Watanabe K., Taniguchi T. & Kanda H. Direct-bandgap properties and evidence for ultraviolet lasing of hexagonal boron nitride single crystal. Nat. Mater. 3, 404–409 (2004). 15156198 10.1038/nmat1134

