# Supplementary material for: Subdiffractional focusing and guiding of polaritonic rays in a natural hyperbolic material
Source: Nat Commun. 2015 Apr 22;6:6963. doi: 10.1038/ncomms7963 (PMC4421822; doi:10.1038/ncomms7963)
Supplement: Supplementary Information — Supplementary Figures 1-5, Supplementary Notes 1-2 and Supplementary References. [file ncomms7963-s1.pdf]

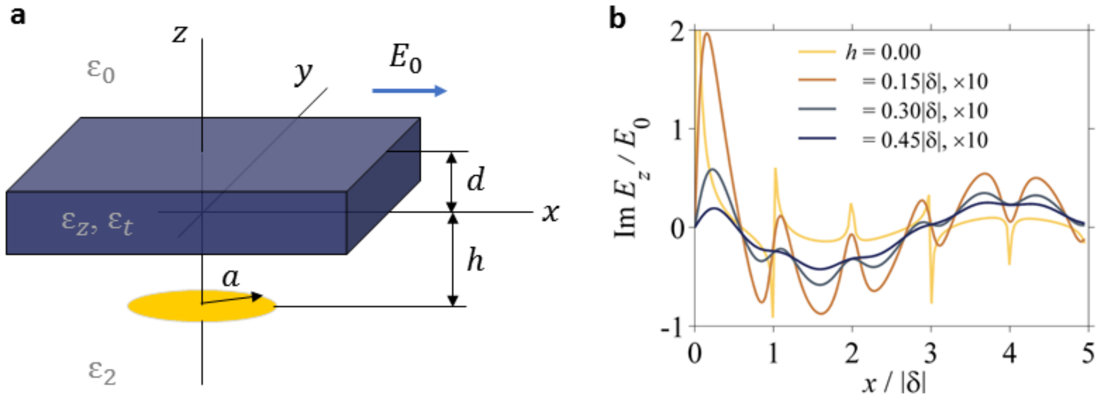

Supplementary Figure 1 | Theoretical model and the electric field profile it predicts. a. The schematics of the model. **b.** The imaginary part of the z-component of the electric field just below the top surface of the slab along the $x > 0$ semi-axis for fixed $a = d = 0.5\delta$ and different h/δ (higher h corresponds to smoother curves; the vertical scale of last three is magnified tenfold.)

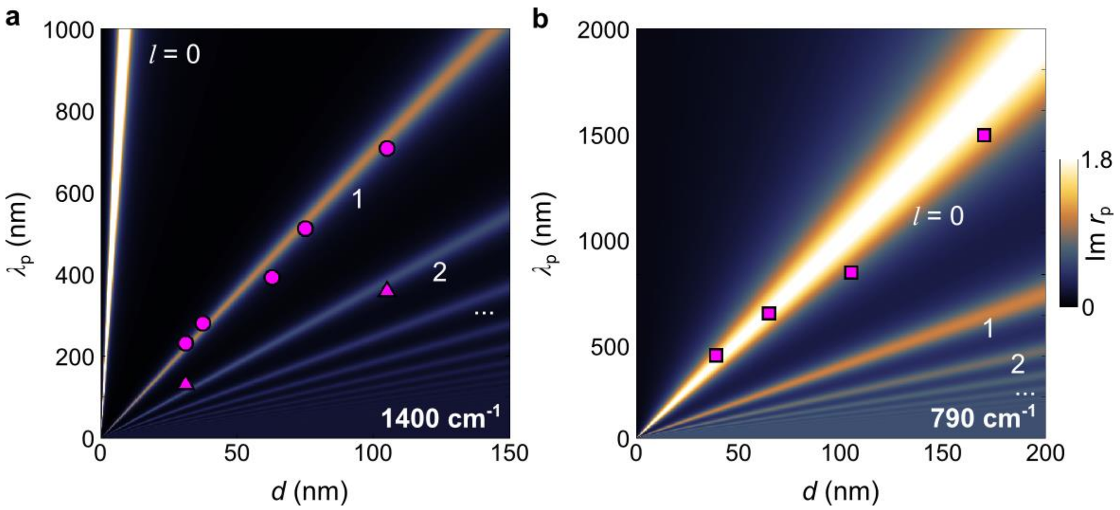

Supplementary Figure 2 | Thickness dependence of the guided wave dispersion. a, The false color plot of the imaginary part of the reflectivity at frequency 1400 cm^{-1} inside the upper (Type II) stop-band of hBN. Experimental data for l = 1 and 2 modes are shown by dots and triangles. **b,** A similar plot for frequency 790 cm^{-1}, which is inside the lower (Type I) stop-band. Here only l = 0 mode has been detected.

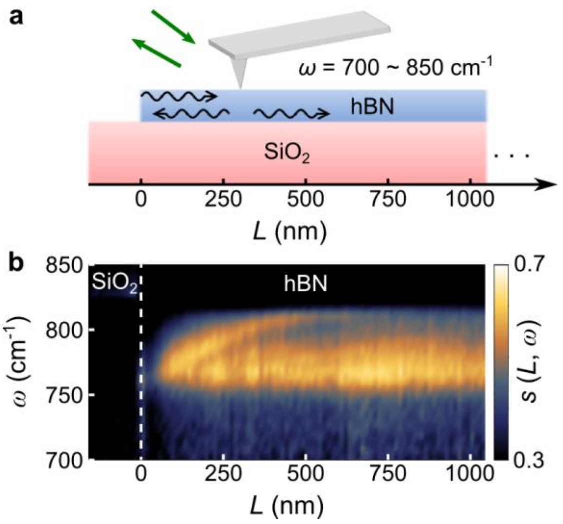

Supplementary Figure 3 | Nano-FTIR study of guided waves in the lower stop-band (Type I spectral region, ω = 746 – 819 cm^{-1}). Since no monochromatic lasers are available in this spectral region, the measurements in the lower stop-band were performed by means of the Fourier transform IR nano-spectroscopy (nano-FTIR) technique[5]. **a,** Schematic of the experiment. A line scan starts from a region of unobscured SiO_2 substrate ($L < 0$) and extends in the region where the hBN crystal resides on SiO_2 ($L > 0$). The green arrows indicate incident and back-scattered broadband IR beam. The black arrows represent the polariton guided waves launched by the tip and reflected by the $L = 0$ edge. **b,** We took near-field spectra at every pixel along the scanning line (**a**) and then obtained the 2D scattering amplitude map $s(L, \omega)$ for a 105-nm-thick hBN crystal on the SiO_2 substrate. The spectra are normalized to Au reference. The dashed line indicates the edge of the crystal. At the hBN side, this map shows the characteristic maxima (interference fringes) created by polariton resonances. The distance of the fringes from the edge increases with the IR frequency ω. This trend is exactly opposite to what we observe in the Type II hyperbolic spectral region, 1350 – 1550 cm^{-1} (see Figs. 4 and 5 of the main text and ref. 5). It implies that the group velocity of the guided waves in the lower stop-band is negative, in accord with its designation as the Type I hyperbolic region. The data points (squares in Fig. 4b of the main text) extracted from ω = constant in this panel, are in a good agreement with the calculated dispersion of $l = 0$ mode (solid line in Fig. 4b of the main text).

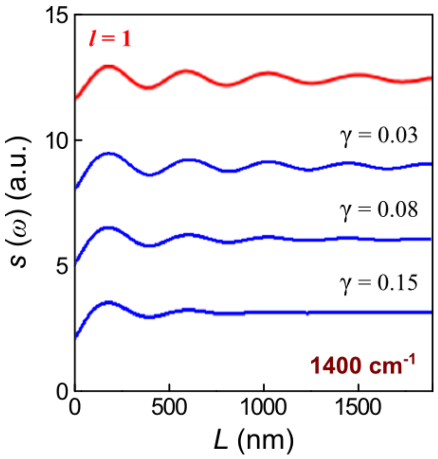

Supplementary Figure 4 | Estimates of the loss factor. To estimate the loss factor from the imaging data shown in from Fig. 5g of the main text, we carried out simulations of the damped sinusoidal line traces with different trial loss factors γ following ref. 5. From these simulations we concluded that the loss factor for the l = 1 mode in Fig. 5g is roughly $\gamma \sim 0.03$. Red trace: contribution of the l = 1 guided wave to the measured scattering amplitude at $\omega = 1400$ cm^{-1} (same as the γ trace in Fig. 5g of the main text). Blue traces: results of the simulation done following ref. 5 for trial loss factors $\gamma = 0.03$, 0.08, and 0.15.

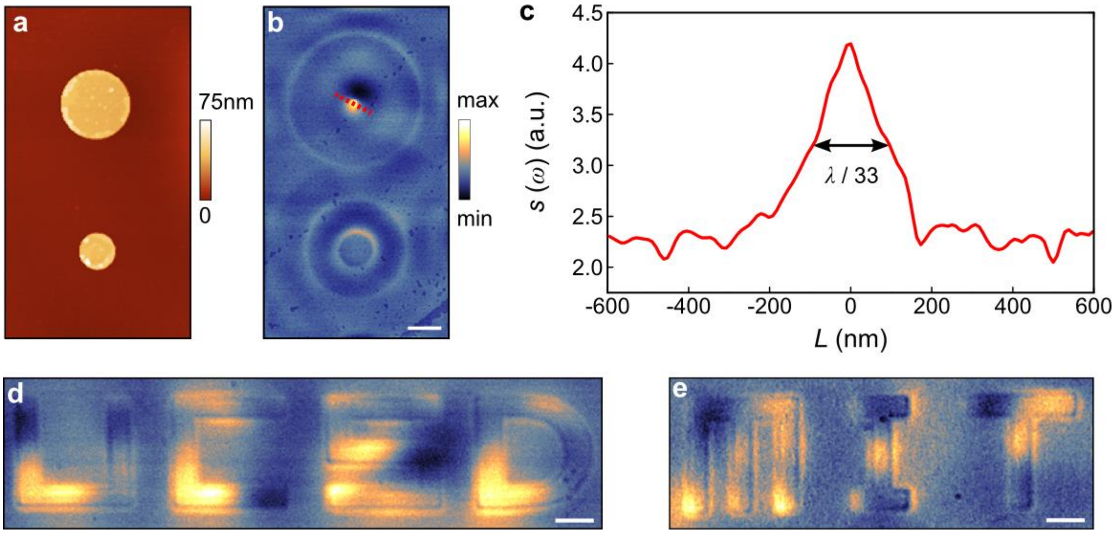

Supplementary Figure 5 | Supplementary s-SNOM images. We achieved small focal spots (FWHM: 185 ~ 210 nm) in all our devices with hBN thickness up to 1050nm. **a,** The AFM image of Au disks of diameter 1μm (top) and 2μm (bottom) on SiO_2/Si substrate before the hBN transfer. **b,** Near-field amplitude image obtained on the top surface of a 1050-nm-thick hBN slab after transferred on (**a**). IR frequency: $\omega = 1541$ cm^{-1}. Remarkably, this device gives a $\lambda/33$ focal spot with a focal distance of $\lambda/6$ (1050 nm, hBN thickness). **c**, Line profile of taken along the red dotted line in (**b**). The black double-arrow indicates the FWHM measured as the focal spot size. In addition to the Au disks, we have prepared and imaged samples with more complicated shapes, for example, in the form of nano-lettered logos of our research institutions (**d**, **e**) at ω = 1610 cm^{-1} (Supplementary Figs. 5d-e). We also see an approximately 1:1 copy of the pattern underneath hBN, in agreement with the theoretical picture of the directional propagation of the polariton rays almost normal to the surface, Fig. 3d (bottom) of the main text. Interestingly, some parts of the image have higher intensity than others. The origin of these intensity variations may be studied in future experiments. Scale bar: 1μm.

Supplementary Note 1: Theoretical model for focusing by a slab of a hyperbolic material

The problem we want to solve is computing the distribution of the electric field $\vec{E}$ inside a hyperbolic medium (HM) slab when a metallic disk is positioned next to its surface and the entire system is subject to a uniform in-plane field $\vec{E}_0$. The schematics of the model are shown in Supplementary Fig. 1a.

We assume that the slab with the axial permittivity $\operatorname{Re} \varepsilon_z > 0$ and the tangential permittivity $\operatorname{Re} \varepsilon_t < 0$ occupies the region $0 < z < d$ and refer to it as medium 1. The media above and below the slab have isotropic permittivities ε_0 and ε_2, respectively. To simplify the analysis, we assume that the metallic disk is infinitely thin. Of course, disks used in the actual experiments are of a finite thickness. To approximately account for the finite thickness, we choose the position of the disk in the model to be some distance h away from the bottom surface of the slab, i.e., in the $z = -h$ plane, which is inside medium 2. We choose the center of the disk to reside on the z-axis. The external field $\vec{E}_0$ is taken to be in the x-direction.

If the disk radius a is much smaller than the free-space photon wavelength, it is sufficient to use the quasi-static approximation for the electric field,

$$\vec{E} = -\nabla\Phi_j \,, \tag{1}$$

where the scalar potentials Φ_j obey the following conditions. Potentials Φ_0 and Φ_2 satisfy the Laplace equation in media 0 and 2 while potential Φ_1 satisfies the equation

$$[\varepsilon_z \partial_z^2 + \varepsilon_t(\partial_x^2 + \partial_y^2)]\, \Phi_1 = 0 \tag{2}$$

inside the slab. The boundary conditions are: $\Phi_2 = 0$ on the disk, $\Phi_j \simeq -E_0 x$ at infinity for all j, and

$$\varepsilon_z \partial_z \Phi_1 = \varepsilon_j \partial_z \Phi_j \tag{3}$$

at the two interfaces. Although the exact analytical solution of this problem does not seem to be possible (cf. ref. 1), an approximate one can be derived based on the solution for a metallic disk inside an *infinite* isotropic medium:

$$\phi(x, y, z) = -\frac{2}{\pi} E_0 x \left(\frac{\sinh \eta}{\cosh^2 \eta} + \arctan \sinh \eta \right), \tag{4}$$

$$\cosh \eta = \frac{1}{2a} \sqrt{\left(\sqrt{x^2 + y^2} + a\right)^2 + z^2} + \frac{1}{2a} \sqrt{\left(\sqrt{x^2 + y^2} - a\right)^2 + z^2} \,. \tag{5}$$

(Here the center of the disk is at the origin of the coordinate frame.) To verify that ϕ is indeed the solution, one can check that ϕ satisfies the Laplace equation, vanishes on the disk, and behaves as

$$\phi(x, y, z) \simeq -E_0 x + \frac{p_x x}{(x^2 + y^2 + z^2)^{\frac{3}{2}}}, \quad p_x = \frac{4}{3\pi} E_0 a^3 \tag{6}$$

at large distances. Quantity p_x can be recognized as the dipole moment acquired by the disk. At the disk edge potential ϕ has a square-root singularity; hence, the electric field has an inverse square-root divergence. These properties are familiar from classical electrostatics.

In order to make use of Supplementary Equations (4)-(5) our first step is to generalize them for the case of an anisotropic uniaxial medium. As obvious from Supplementary Equation (2), this can be achieved by rescaling of the axial coordinate: $z \to (-i \tan\theta)\, z$, where

$$\tan\theta = i\,\frac{\sqrt{\varepsilon_t}}{\sqrt{\varepsilon_z}} = \frac{\delta}{2d}, \quad \delta = 2d \tan\theta \tag{7}$$

(cf. equations (2) and (3) of the main text). If the imaginary parts of the permittivities are negligibly small, the rescaling factor is pure imaginary. This changes the nature of the solution qualitatively. It becomes possible for the arguments of the square roots to vanish not only at the edge but also at a set of points in space whose coordinates satisfy the equation

$$\sqrt{x^2 + y^2} = \pm a \pm z \tan\theta\,. \tag{8}$$

This set of points is a union of two cones of the opening angle $|\theta|$ coaxial with the disk and passing through its edge. The apices of the cones are located at $\mathrm{z} = \pm \mathrm{a} \cot\theta$. The electric field has the inverse square-root divergence on these cones, which is consistent with the physical picture of hyperbolic phonon polaritons (HP^2) launched predominantly at the disk edge. Similar high field intensity cones have been previously studied in plasma physics and dubbed "resonance cones"[2]. The apices of the cones act as focal points where the rays launched at the edge intersect. At these points the singularity of the electric field is even stronger. From Supplementary Equations (4)-(5) we deduce that the z-component of the electric field in the "focal plane" behaves as

$$\frac{E_z}{E_0} \propto \frac{ix}{x^2 + y^2} \tag{9}$$

if the damping is absent and there is no gap between the disk the the slab, $h = 0$. This inverse-distance divergence does not have an intrinsic scale. However, one can show that if the imaginary parts of the permittivities are not neglected and/or h is nonzero, then the z-component of the field vanishes rather than diverges at $x = y = 0$ and

that it attains its maximum at positions $(\pm x_m, 0)$, where

$$x_m = \sqrt{\frac{1+\sqrt{5}}{8}}\,(2h + \mathrm{Im}\,\delta)\,, \tag{10}$$

see Supplementary Fig. 1b. Parameter x_m is one possible measure of the focal spot size. We will discuss numerical estimates of this parameter for hexagonal boron nitride (hBN) and compare them with our experimental results shortly below.

Our next step is to use Supplementary Equation (4) valid for an unbounded medium as a building block for constructing a solution for the case of a finite-thickness slab. We follow the procedure standard in the method of images and consider an approximate solution as follows:

$$\begin{aligned} \Phi_1 \quad &= \quad -\sum_{n=1}^{\infty} r_0^n r_2^{n-1} \phi\left(x, y, -h - \frac{i\delta}{2d} z + in\delta\right) \\ &+ \quad \sum_{n=0}^{\infty} r_0^n r_2^n \phi\left(x, y, h - \frac{i\delta}{2d} z - in\delta\right), \end{aligned} \tag{11}$$

where

$$r_j = \frac{\varepsilon_j - \sqrt{\varepsilon_z}\sqrt{\varepsilon_t}}{\varepsilon_j + \sqrt{\varepsilon_z}\sqrt{\varepsilon_t}} \tag{12}$$

is the reflection coefficient at interface of the slab and medium $j = 0, 2$. In the absence of damping in the system, these coefficients are complex numbers of unit modulus, i.e., they are phase factors. The relation of these phases to the parameter α used in the main text (equation (5)) is

$$r_0 r_2 = e^{-2\pi i \alpha}. \tag{13}$$

The top line in Supplementary Equation (11) represents the scalar potential created by an infinite series of fictitious images above the slab. The sum on the bottom line contains the potential of the disk and of another infinite series of images below it. Although the complex amplitudes of the consecutive image terms, e.g., $r_0^n r_2^n$ do not decay by the absolute value, images with higher n are more distant from the slab. As a result, they produce progressively weaker potential inside the slab, which ensures convergence of the series. By construction, the potential given by Supplementary Equation (11) meets the boundary conditions at the slab surfaces. The image terms modify the asymptotical value of the electric field. However, if we add the normalization factor $N = (1 - r_0 r_2)/(1 - r_0)$, we bring it back to E_0. This way, the boundary condition at infinity will also be satisfied. Unfortunately, the potential given

by Supplementary Equation (11) violates the equipotential boundary condition on the disk and there is no simple way to remedy that. Similar difficulty appears in the classic electrostatic problem of a circular parallel-plate capacitor of finite radius[1]. The potential distribution in such a capacitor is not equal to the sum of the potentials created by each charged plate in isolation. However, it does look qualitatively similar. In particular, the square-root singularity at the edge is the universal feature, which must also be exhibited by the exact solution. We surmise that in our problem the main inaccuracy of Supplementary Equation (11) is the strength of the square-root edge singularity. Otherwise, our approximate solution for Φ_1 should capture the qualitative aspects of the radiation cones emanating from the edges correctly. Note that the influence of the s-SNOM tip is not considered in our theory since its effect is expected to be only quantitative, and not qualitative. As is well established in literature, the qualitative features of the near-field contrast are adequately described assuming that the near-field signal registered by the tip is proportional to the electric field just above the surface of the sample[3-6].

To get the desired electric field component E_z, we take the derivative of Φ_1 with respect to z, which we can easily do analytically. The results are plotted in the false color in Fig. 3c of the main text for different sizes of the disk. These plots depict the case $h = 0$, i.e., no gap between the disk and the slab. The optical constants used in the calculation are $\varepsilon_0 = 1$, $\varepsilon_t = -3.50 + 0.15i$, $\varepsilon_z = 2.80 + 0.0005i$, and $\varepsilon_2 = 1.39 + 0.015i$, which is appropriate for hBN slab and SiO_2 substrate at frequency ω = 1515 cm^{-1}. Using Supplementary Equations (7), (12), and (13), we find $\tan\theta \approx -1.12$ and $\alpha \approx -0.23$ (both are nearly pure negative). As one can see from Fig. 3c of the main text, the method of images accounts for the multiple reflections of HP^2 inside the slab that produce the concentric rings of high intensity electric field on the top surface. The rings' radii are given by equation (4) of the main text,

$$r_n = \left| a + \left(n - \frac{1}{2}\right)|\delta| \right|, \quad n = 0, \pm1, \pm2, \dots \tag{14}$$

(The absolute value of δ is taken in this formulas because δ can and indeed is negative in the cited example.) It is clear from the derivation that the described model has a scaling property: if a and δ are both multiplied by the same factor, the electric field as a function of dimensionless coordinates: x/a and $z/|\delta|$ does not change. Accordingly, the qualitative aspects of the electric field distribution are controlled by the dimensionless ratio $a/|\delta|$. For example, the n-index of the smallest ring is the integer closest to $1/2 - a/|\delta|$. The evolution of the strength and relative arrangement of the hot rings is illustrated in Fig. 3b of the main text for several $a/|\delta|$ ratios and also in Fig. 3d of the main text for several θ. In all these examples the smallest ring has the radius $r_0 = \left| a - \frac{1}{2}|\delta| \right|$.

Particularly interesting is the first case where this ring shrinks to a point, $r_0 = 0$. In the other examples sharp focal points are found inside the crystal, at the intersections of the launched and reflected resonance cones. For the quoted above values of the permittivities and the slab thickness $d = 395$ nm we get $\delta = 2 \times 395\ \text{nm} \times i\sqrt{-3.50 + 0.15i}\ / \sqrt{2.80} = (-880 + 19i)$ nm. Assuming also that the effective disk-slab separation is $h = 25$ nm, one half of the disk physical thickness, we get $x_m \approx 70$ nm from Supplementary Equation (10). Hence, the distance between the maxima of $\operatorname{Im} E_z$ occurring on the opposite sides of the focal spot is $2x_m \approx 140$ nm (Supplementary Fig. 1b). When comparing this estimate with the focal spot size in the experimental images, such as Fig. 2b, one has to keep in mind that the measured quantity is not $\operatorname{Im} E_z$ but the demodulated scattering amplitude $s(\omega)$, which is a certain functional of E_z. Also, in the experiment the incident light has a mixture of different polarizations whereas linear polarization is assumed in the model. These are likely reasons for minor differences between the experimental images and the $\operatorname{Im} E_z$ curves in Supplementary Fig. 1b. For example, the calculated $\operatorname{Im} E_z$ vanishes along the line $x = 0$ but the scattering amplitude measured in the experiment does not exactly vanish along any direction. In practice, our procedure to extract the focal spot size from the images was to take a linear cut along the direction where the signal looked like a simple peak and then take the full width at half-maximum (FWHM) of this peak (Supplementary Fig. 5b). The above number $2x_m \approx 140$ nm gives a rough theoretical estimate of the focal spot size obtained by this procedure. It is in fact in agreement with the FWHM of 185 ~ 210 nm determined for the s-SNOM images (Figs. 2b in the main text and Supplementary Fig. 5b).

To make connection with the second part of the article we use the fact that the real-space distribution of the scalar potential can be alternatively represented by a two-dimensional (2D) Fourier integral. After some lengthy but straightforward calculation, it is possible to show that Supplementary Equation (11) implies that the electric field just above the top surface of the slab can be written as

$$E_z(x, y, d + 0) = 8E_0 a^3 \int \frac{dk_x}{2\pi} \int \frac{dk_y}{2\pi}\, e^{ik_x x + ik_y y}\, t(k_t)\; ik_x \left[\frac{\sin k_t a}{(k_t a)^3} - \frac{\cos k_t a}{(k_t a)^2}\right], \qquad (15)$$

$$t(k_t) = \frac{\sin \pi\alpha}{\sin\left(\pi\alpha - \frac{1}{2} k_t \delta\right)}\, e^{-k_t h}, \qquad k_t = \sqrt{k_x^2 + k_y^2}\,. \qquad (16)$$

The product of the last two terms in the integrand of Supplementary Equation (15) represents the amplitude of the Fourier harmonics with momentum (k_x, k_y). The absolute values of these amplitudes exhibit slow power-law decay $\sim k_t^{-1}$. Accordingly, the Fourier spectrum of the electric field induced in our system is very broad. The inverse square-root divergence of the field at the “hot rings” arises from the constructive interference among the harmonics of this broad spectrum. The

dominant contribution comes from the harmonics with momenta near the discrete set of values

$$k_{t,l} = \frac{2\pi}{\delta}(l + \alpha)\,, \tag{17}$$

(same as equation (5) of the main text) at which function $t(k_t)$ exhibits pole singularities. These are the momenta of the guided modes of the slab. This is why we made a statement in the main text that the image formed on the top surface of the slab can be viewed as a coherent superposition of multiple guided waves launched by the disk.

It is worth pointing out that function $t(k_t)$ has the meaning of the transmission coefficient of HP^2 between the metallic disk and the top surface of the slab. (More precisely, $t(k_t)$ a transmission coefficient multiplied by the momentum-independent factor N, mentioned above, that enforces the normalization $t(0) = 1.$) This transmission coefficient includes the factor $e^{-k_t h}$ due to evanescent decay across the vacuum gap and the Fabry-Pérot-like resonant transmission factor due to the free propagation of HP^2 inside the HM slab. If a slab were made from a non-HM, parameters δ, α, and so the guided wave momenta $k_{t,l}$ defined by Supplementary Equation (17) would be predominantly imaginary. As a result, instead of the resonant transmission HP^2 going through the slab would suffer yet another exponential decay.

Finally, let us now discuss another interesting effect, which may perhaps be verified by future experiments. The finite thickness of real metallic disks is crudely accounted for in our model by choosing a nonzero disk-slab distance h. This parameter enters the last exponential factor in Supplementary Equation (16) thereby imposing a soft momentum cutoff $k_t \sim h^{-1}$ in the integral of Supplementary Equation (15). Accordingly, the characteristic number of the guided modes that can effectively contribute to the image formation on the opposite surface of the slab becomes limited to

$$|l_{\max}| \sim \frac{|\delta|}{2\pi h}. \tag{18}$$

Therefore, as h increases, the high-l guided waves are progressively eliminated. Eventually, at $h \gg |\delta|$, the amplitude of even the lowest-momentum $l = 0$ mode becomes exponentially small. However, since the higher-order modes are suppressed even more, this mode dominates the spatial oscillations of the field. The corresponding period $2\pi/\left|k_{t,0}\right| = |\delta/\alpha|$ is in general incommensurate with and several times larger than $|\delta|$, the repeat distance of the "hot rings" at $h = 0$.

In Supplementary Fig. 1b we illustrate these trends by numerical simulations of the electric field profile in the "surface-focusing" case $|a/\delta| = 0.5$ for several different h, with other parameters kept the same as in Fig. 3c of the main text. We choose to plot the imaginary part of the electric field, i.e., the field component that is $\pi/2$-out of phase with respect to the external field E_0. (The real part, i.e., the in-phase component, shows a similar behavior.) Two facts that agree with the analytical picture

are readily apparent from Supplementary Fig. 1b. First, at nonzero disk-slab separation the magnitude of the field can be much smaller than for disk right next to the slab. Actually, to show $h > 0$ curves clearly we have to scale them by the factor of ten in Supplementary Fig. 1b. Second, as h grows, the sharply peaked extrema of the electric field producing the equidistant series of “hot rings” separated by $|\delta|$ gradually transform into smooth sinusoidal oscillations of a larger period. This period is approximately four times $|\delta|$, in agreement with the expected result $|\delta/\alpha|$. (Recall that $\alpha \approx -0.23$ in this example.)

Supplementary Note 2: Guided wave dispersion and its thickness dependence

The thickness dependence of the $l = 0$ guided waves in hBN has been studied in our previous work[5]. Here we first summarize the procedure and then apply it to analyze the newly discovered $l \neq 0$ guided wave modes.

The momenta $k_{t,l}$ of the guided waves given by Supplementary Equation (17) have been defined above as the poles of the transmission coefficient $t(k_t)$. These momenta are complex, which simply means that the guided waves exhibit finite damping. As usual, the real part of each $k_{t,l}$ determines the wavelength $\lambda_{p,l} = 2\pi \,/\, \mathrm{Re} k_{t,l}$, while the ratio of the imaginary and real parts specifies the loss factor $\gamma = \mathrm{Im}\, k_{t,l} \,/\, \mathrm{Re}\, k_{t,l}$. Instead of the transmission coefficient $t(k_t)$, one can examine the surface reflectivity

$$r_p(k_t) = \frac{-r_0 + r_2 e^{i\delta k_t}}{1 - r_0 r_2 e^{i\delta k_t}} \tag{19}$$

because the poles of the two functions coincide, see Supplementary Equations (13), (16), and (19). However, the reflectivity becomes more useful for the second method of determining the mode dispersions. Namely, the imaginary part of the reflectivity as a function or real momentum is always positive and if the loss factor is small enough, it has sharp maxima at $\mathrm{Re}\, k_{t,l}$. Finding such maxima is easy to implement numerically. For the case $l = 0$, this method[7] becomes advantageous at low momenta where the quasi-static approximation becomes inaccurate and Supplementary Equation (19) has to be replaced by a more complicated expression based on the full Fresnel formulas. However, higher-order modes studied here possess rather high momenta, so either method can be used.

Let us now discuss how this procedure applies specifically to the thickness dependence of the guided wave spectra in hBN. The false color plot of $\mathrm{Im}\, r_p$ at a representative IR frequency ω = 1400 cm^{-1} is shown in Supplementary Fig. 2. It has been calculated using the permittivity functions of hBN and SiO_2 from ref. 5 and 8 as input parameters. The bright lines in this plot give $\lambda_{p,l}$. Whereas Supplementary Equation (17) predicts a strictly linear dependence of $\lambda_{p,l}$ on crystal thickness d, the bright lines in Supplementary Fig. 2 exhibit a slight curvature at small momenta. Actually, at experimentally relevant momenta the difference between the two methods of determining $\lambda_{p,l}$ is negligible. These experimental results are shown by dots and

triangles in Supplementary Fig. 2a. They have been found using the Fourier-transform method described in the main text. The measurements were done on several different hBN specimens. The thickness d for each of the specimen was measured via the AFM topography simultaneously with the IR images. Matching the symbols with the nearby lines makes it possible to identify the modes as l = 1 for the dots and l = 2 for the triangles. Within the shown range of d, the measured wavelengths of these modes scale linearly with the hBN thickness, in accord with the theory. Supplementary Figure 2b shows another set of results for the frequency $\omega = 790\,\mathrm{cm}^{-1}$, which belongs to the lower stop-band (Type I region). Here the polaritons have a negative group velocity but they obey the same linear scaling with d as in the upper band, which is again in agreement with the theory.

Supplementary References

1. Chew, W. C. & Kong, J. A., Microstrip Capacitance for a Circular Disk Through Matched Asymptotic Expansions, *SIAM J. Appl. Math.* **42**, 302–317 (1982).
2. Fisher, R. K. & Gould, R. W. Resonance cones in the field pattern of a short antenna in anisotropic plasma. *Phys. Rev. Lett.* **22,** 1093-1095 (1969).
3. Huber, A., Ocelic, N., Kazantsev, D. & Hillenbrand, R. Near-field imaging of mid-infrared surface phonon polariton propagation. *Appl. Phys. Lett.* **87**, 081103 (2005).
4. Huber, A. J., Deutsch, B., Novotny, L. & Hillenbrand, R. Focusing of surface phonon polaritons. *Appl. Phys. Lett.* **87**, 081103 (2005).
5. Dai, S. *et al*. Tunable phonon polaritons in atomically thin van der Waals crystal of boron nitride. *Science* **343**, 1125-1129 (2014).
6. Alonso-González, P. et al. Controlling graphene plasmons with resonant metal antennas and spatial conductivity patterns. *Science* **344**, 1369 (2014).
7. Zhang, L. M. *et al*., Near-field spectroscopy of silicon dioxide thin films. *Phys. Rev. B* **85**, 075419 (2012).
8. Cai, Y., Zhang, L., Zeng, Q., Cheng, L. & Xu, Y., Infrared reflectance spectrum of BN calculated from first principles. *Solid State Commun.* **141**, 262 (2007).
